# Family-centred service in paediatric acquired brain injury rehabilitation: Bridging the gaps

**DOI:** 10.3389/fresc.2022.1085967

**Published:** 2022-12-23

**Authors:** Taylor Jenkin, Vicki A. Anderson, Kate D'Cruz, Adam Scheinberg, Sarah Knight

**Affiliations:** ^1^ Neurodisability & Rehabilitation, Murdoch Children's Research Institute, Melbourne, VIC, Australia; ^2^ Melbourne School of Psychological Sciences, The University of Melbourne, Melbourne, VIC, Australia; ^3^ Brain and Mind, Murdoch Children's Research Institute, Melbourne, VIC, Australia; ^4^ Psychology Service, The Royal Children's Hospital, Melbourne, VIC, Australia; ^5^ Summer Foundation, Melbourne, VIC, Australia; ^6^ Department of Paediatrics, The University of Melbourne, Melbourne, VIC, Australia; ^7^ Victorian Paediatric Rehabilitation Service, The Royal Children's Hospital, Melbourne, VIC, Australia

**Keywords:** acquired brain injury (ABI), family-centred service, paediatric rehabilitation, family-centred care, rehabilitation

## Abstract

**Background:**

Children and adolescents who sustain an acquired brain injury (ABI) can experience acute and ongoing difficulties in a range of cognitive and functional domains, and their families often experience significant life changes and challenges. Family-centred service is therefore considered best practice in paediatric ABI rehabilitation. Despite widespread acceptance of family-centred service in this context, recent literature indicates that family needs are often unrecognised and unmet following paediatric ABI. Although family-centred service was introduced in the field of developmental disability over five decades ago, there remains a lack of clarity about how this approach is implemented in practice. Additionally, limited literature has discussed the implementation of family-centred service in paediatric ABI rehabilitation despite key differences between ABI and developmental disability, including nature and timing of onset, rehabilitation foci, and impacts on families.

**Aims:**

In this review, we aim to: (i) outline common sequelae of paediatric ABI with a focus on family outcomes; (ii) summarise paediatric rehabilitation and highlight opportunities for family support and involvement; (iii) discuss and synthesise literature across paediatric ABI rehabilitation and family-centred service to highlight gaps in knowledge and practice; and (v) identify clinical implications and future research directions.

**Conclusions:**

There is a clear need for greater clarity and consensus regarding the implementation of family-centred service in paediatric ABI rehabilitation. This review highlights the importance of providing professional development opportunities for clinicians to increase competency in practising in a family-centred manner, and opportunities to actively involve, empower and support families within rehabilitation. This review also emphasises the importance of services implementing relevant supports to address family needs where possible and developing clear referral pathways so that families can access further support elsewhere when needed.

## Introduction

Paediatric acquired brain injury (pABI) is a leading cause of death and acquired disability in children and adolescents, and refers to a brain insult sustained after birth (the term “children” will henceforth be used to refer to children and adolescents) (1–3). pABI can arise as a result of a range of causes, including traumatic brain injury (TBI), cerebrovascular accident (stroke), brain tumour, cerebral anoxia (oxygen deprivation), or infections such as encephalitis or meningitis (3–5). This wide range of pABI causes has led to difficulty in accurately estimating incidence. Hospital emergency department (ED) presentations for TBI, the most common form of pABI, have been reported to be approximately 2008 per 100,000 children (6). The consequences of pABI are complex and can profoundly impact children and their families. Family-centred services are widely accepted as best practice and emphasise the importance of supporting and involving families in pABI rehabilitation (7–10). While there are varying approaches to the delivery of family-centred service (9, 11, 12), evidence suggests that family needs are often unrecognised and unmet in rehabilitation following pABI (13–16).

This review aimed to: (i) outline common sequelae of pABI with a focus on family outcomes; (ii) summarise paediatric rehabilitation and highlight opportunities for family support and involvement; (iii) discuss recent literature regarding family-centred services; (iv) summarise literature across pABI rehabilitation and family-centred service to highlight gaps in knowledge and practice; and (v) identify clinical implications and future research directions.

## Developmental considerations in paediatric ABI

Children who sustain an ABI can experience a range of acute and persisting difficulties in cognitive, academic, emotional, and behavioural functioning, which vary in severity from mild to severe and are associated with reduced participation and quality of life (3, 17, 18). In considering the impacts of pABI on the lives of children, it is also important to recognise the developmental context in which a pABI occurs. Childhood and adolescence are periods of rapid and dynamic brain development, with maturation continuing into the early 20s (19, 20). pABI can therefore disrupt acquired skills and derail ongoing skill development (21, 22), with children with ABIs “growing into” their deficits, and falling increasingly further behind their age peers over time (21–25). Thus pABI can cause long-term disability that persists into adulthood and associated lifetime consequences (3, 26).

### Impact on families

Given the potentially significant acute and long-term impacts of pABI on children, it is unsurprising that families can also experience a dynamic constellation of changes and challenges following pABI. The sudden and unforeseen onset of these insults can contribute to acute and long-term injury-related distress, anxiety, reduced quality of life, reduced wellbeing, and caregiving burden in family members (14, 27–34). Family functioning and dynamics can also change as family members adapt their roles and responsibilities to adjust to a “different life rhythm” (27, 35, 36). While most families adapt to caring for a child with pABI over time (29, 33, 37–39), deterioration in family functioning may continue for several years, particularly for families of children with more severe injuries (29, 40–43). Several interventions have been developed to support families in adapting to life following paediatric ABI and coping with trauma. For example, Family Forward (31, 44) is delivered to families during inpatient rehabilitation and combines family counselling and multifamily groups to support family adaptation and coping. Stepping Stones Triple *P* Plus Acceptance and Commitment Therapy (SSTP + ACT) (45, 46) also addresses parent coping through an acceptance and commitment therapy approach, coupled with parenting skills training.

The developmental context of pABI relates not only to the development of the injured child, but to the development of the family. Expectations and hopes for the future often change when a child sustains a brain insult, after which families may lack a “road map” to anticipate the future (47–51). Parents and caregivers may grieve the loss of a “normal” future for their child and family, as well as the loss of the child they had known (14, 35, 52, 53). This loss may differ from that experienced by families of children with congenital or developmental disabilities which are present “from the beginning” of a child's life (54). The sudden, unforeseen, and often traumatic onset of pABI has important implications for families' support needs and rehabilitation.

The outcomes of children with pABI and their families are bi-directional and children's outcomes are closely associated with family functioning and the family's capacity to meet the changing needs of the child (39, 55–59). Taylor and colleagues (60) found a reciprocal relationship between parent distress and behaviour problems of children with moderate TBI, and others have found that better family functioning is predictive of better long-term outcomes for children (61, 62). These findings align with Family Systems Theory, which posits that each member of a family impacts all others, and the wellbeing of family members influences their capacity to facilitate child development (7, 63–65). Evidence for child-family interactions in pABI recovery highlights the need for rehabilitation to focus on the child *and* their family, not only to facilitate family adjustment and outcomes but also to enable families to better support the child with pABI.

## Contemporary approaches to rehabilitation for paediatric ABI

pABI can give rise to complex patterns of acute and persisting deficits and needs. Many children with moderate to severe ABIs therefore require specialist rehabilitation (3, 4). Rehabilitation refers to “a set of interventions designed to optimize functioning and reduce disability in individuals with health conditions in interaction with their environment,” and aims to enable participation in meaningful life roles (66). Given the developmental context, pABI rehabilitation seeks to both target the effects of the pABI and support progress towards normative developmental milestones (67, 68). Additionally, as the impacts of pABI on children may not fully manifest for many years, rehabilitation requires an understanding of children's changing needs and a focus on providing ongoing support (3, 69–73). This is particularly relevant during transition periods such as the transition from home to school or from childhood to adolescence, at which point the functional impacts of pABI often become most apparent as developmental demands increase (4, 25, 74).

While early rehabilitation models were predominantly unimodal or domain-specific, there have been recent advances towards more holistic, context-sensitive, and integrated care (58, 67). To facilitate this approach, interdisciplinary rehabilitation is considered best practice and requires integration of disciplines’ efforts through collaboration and communication, such that team members function synchronously to work towards a superordinate goal that transcends discipline boundaries, with a shared understanding of each other's knowledge and methods (75–80). Strengths-based approaches are also gaining increasing recognition in pABI rehabilitation (81–83). This orientation emphasises individuals' and families' resilience and strengths, and recognises opportunities for healing, growth, and hope for the future following pABI (84).

### Shifting the focus from impairment to functioning

Traditional views of childhood disability and rehabilitation have been strongly influenced by the biomedical model which focuses on “fixing” health conditions or disability through intervention provided by health professionals (85, 86). In 2001, the World Health Organization published the International Classification of Functioning (ICF) (87), a biopsychosocial framework that conceptualises health as a person's functioning within their social-ecological context (85, 86). This holistic framework depicts the interconnections between body structure and function, activity, participation, environmental factors, and personal factors, and recognises that no one factor is more important than the others (85). Within childhood disability, the Children and Youth Version of the ICF (88) encourages consideration of factors that are important to children's development, including their participation (i.e., involvement in life situations), activities (i.e., execution of tasks and actions), and environment (i.e., physical, social, and attitudinal environments in which people live)(85, 87, 89). To encourage the implementation of the ICF, Rosenbaum and Gorter (85) formulated six “F-words” (function, family, fitness, fun, friends, and future) to apply when working with children with disabilities and their families. These “F-words” are embedded within the components of the ICF and illustrate a biopsychosocial approach to disability, focusing on children's strengths and what they *can* do, rather than disability-related limitations (85, 90). Within this framework, “family” represents the child's environment or context. Implementation of the “F-words” supports improvements in children's health and functional outcomes, family empowerment and satisfaction, and overall family-centred service (90).

#### Family-centred service

Family-centred service is considered best practice in contemporary pABI rehabilitation (7–9, 91, 92). While family-centred service acknowledges clinicians' knowledge regarding medical conditions and their treatments, this approach emphasises that “*each family is unique*; that the family is the *constant in the child's life*; and that they are the *experts on the child's abilities and needs*” (93). Consistent with a family systems approach, family-centred service is underpinned by an understanding that better family functioning means that families are better equipped to care for their children and facilitate their development (7, 64, 65, 94). It also emphasises the role of the family in care planning, implementation, and evaluation, such that clinicians “work *with* patients and families, rather than just doing *to* or *for* them” (95).

### Family involvement, engagement, and participation in rehabilitation

Family-centred service requires partnership between healthcare providers and families; thus, it is essential to understand what family participation, attendance, and engagement mean in rehabilitation. Parent participation in therapy refers to the actions they take to be actively involved in all stages of their child's care, for example, through sharing information to inform assessment or treatment, or implementing therapeutic exercises at home (96–98). Imms and colleagues (99) further define participation as comprising two elements: attendance and involvement. Given that the term “engagement” is used more commonly than “involvement” in healthcare literature, the term “engagement” will henceforth be used. Attendance refers to “being there” (100), and while this is often considered a behavioural indicator of parents' and caregivers' engagement, attendance alone does not necessarily constitute their engagement (96, 101). Rather, parent engagement is complex and multifaceted, representing a process of “engaging with” and a state of “engaging in” children's therapy (96, 102–104). Engagement “with” therapy is a relational, co-constructed, collaborative process between individuals and clinicians or services, whereas engaging “in” therapy is a state of affective, behavioural and cognitive commitment or investment in therapy that involves “being with what you are doing” (102–105). In a recent constructivist grounded theory study, Phoenix and colleagues (106) explored parent attendance, engagement, and participation in children's developmental rehabilitation services. They highlighted the impacts of parents' ﻿feelings, skills, knowledge, logistics, values and beliefs, and relationships with healthcare professionals on participation, attendance, and engagement, and considered the influence of family composition, service complexity (i.e., number of organisations and professionals involved in the child's healthcare), and health complexity (i.e., child, sibling, and parent physical and mental health). While most of this literature has focused on only parents or caregivers, the concepts of participation, attendance, and engagement can also be applied to other family members.

While limited literature has investigated family participation, attendance, and engagement in pABI rehabilitation, a recently published qualitative study explored family participation in pABI rehabilitation from the perspectives of children and adolescents with pABIs and their parents/caregivers and siblings (107). Findings highlighted how families can be involved in “doing rehabilitation together” with the child or adolescent, including participating in collaborative decision-making, actively supporting the child's or adolescent's input and engagement, and learning through observation and conversations with clinicians. Findings demonstrated that family participation in rehabilitation spans the entire care trajectory, from involvement in early inpatient rehabilitation to supporting the child to engage in rehabilitation activities at home. Importantly, this study highlighted that family participation in rehabilitation is shaped by families' unique lives and contexts and relies on two-way communication and information sharing between clinicians and families.

Given the importance of family participation in rehabilitation and the significant impacts of pABI on families, several family-centred interventions have been developed and implemented in rehabilitation. Cermak and colleagues (108) conducted a systematic review and meta-analysis of parent interventions following paediatric TBI and outlined interventions targeting parent outcomes, including parent-child interaction therapies (109, 110), problem-solving interventions (111, 112), and interventions combining parenting and cognitive behaviour therapy (113). In addition to parent interventions, several other family-centred interventions have been developed in the context of pABI that involve partnering with parents and caregivers to deliver context-sensitive rehabilitation, collaborative goal setting, parent psychoeducation, and family psychosocial support (31, 114, 115). Interventions of this nature enable family participation in rehabilitation and provide much needed support to families following pABI.

### Understanding family needs in rehabilitation

pABI can have significant impacts on families, and protective factors such as interpersonal support, coping skills, and access to knowledge and information can buffer against decrements in family adaptation, burden, and distress (116, 117). While a family-centred approach that involves and supports families is considered best practice in pABI rehabilitation, a recent scoping review revealed that children and young people with pABI and their families report unrecognised or unmet information and emotional support needs across the care trajectory (13). This included the need for individualised information regarding the impact of the child's injury, as well as current and future treatment plans (13, 14). Families may also have high needs for emotional support, particularly following the child's return home (13–15). Greater recognition of the impacts of pABI on the whole family is also needed, as is increased support for siblings (118–120). While social support and peer connection are important for families following pABI, they may require assistance accessing such resources (73, 121). Long-term follow-up and family support in managing the ongoing sequelae of pABI, including behavioural and psychological difficulties is also a consideration (13, 14, 107).

### Collaborative goal setting

Collaborative goal setting, a core component of adopting a family-centred approach in paediatric rehabilitation, is the process by which individual rehabilitation goals are identified and negotiated (122–124). Collaborative goal setting between children, families, and clinicians is considered best practice in paediatric rehabilitation, representing an opportunity for children and families to have input into care planning (11, 125, 126). Through collaboration, meaningful goals can be identified, facilitating rapport, increasing children's and families' engagement and motivation in rehabilitation and enhancing parents' feelings of competency (127–130). There is wide variability in goal setting processes among services (124, 131, 132) and formalised goal setting methods are inconsistently implemented (133, 134). Several researchers have proposed frameworks to promote engagement in goal generation. For example, Prescott and colleagues (135, 136) developed the Client-Centred Goal Setting Practice Framework for goal setting with adults with ABI in community-based rehabilitation, and Pritchard-Wiart and colleagues (137) developed a child-focused approach to goal setting, Enhancing Child Engagement in Goal Setting (ENGAGE). While neither of these frameworks are specific to pABI rehabilitation, both have potential for adaptation in this context.

Limited literature has investigated collaborative goal setting in pABI rehabilitation. Evidence regarding goal setting in the context of childhood disability more broadly has found that meaningful goals are linked to greater engagement and motivation in therapy, improved rehabilitation outcomes, and enhanced family-clinician partnership (130, 138–141). When children are involved in rehabilitation goal setting, they feel valued and heard (142), but when children's preferences are not considered, they may feel “powerless and depersonalized” (143–145). Two recent qualitative studies explored experiences of goal setting in outpatient pABI rehabilitation from the perspectives of rehabilitation clinicians (146) and children and their caregivers (147). Findings emphasised the importance of clinicians, children, and parents/caregivers collaboratively generating meaningful rehabilitation goals, and highlighted the active role of clinicians in educating young people and their parents about goal setting and supporting their engagement in goal setting. The findings of these two qualitative studies suggest that collaborative goal setting provides important opportunities for young people with pABI and their families to have input into, and direct, their own healthcare, thus aligning with a family-centred approach to rehabilitation.

## Discussion

### Gaps in knowledge and practice

#### Lack of consensus around the definition of family- centred service

Although the concept of family-centred service was introduced over five decades ago, a consensus definition has not yet been achieved and there remains a lack of clarity about how this approach should be implemented in practice (9, 11, 12). Many definitions of family-centred service have been proposed and several general principles are common among these definitions, including the open exchange of information with families, respect for family differences and care preferences, partnership and collaboration with families in decision-making at the level they choose, and the provision of care in the context of the family and their community (9). In recognition of the lack of a universal model of family-centred service, Kokorelias and colleagues (10) conducted a scoping review of family-centred models of care in paediatric and adult healthcare. A common goal of the 55 included models was the development and implementation of care plans within the context of the family. Key components to facilitate family-centred service were also identified, including: collaboration and communication among the healthcare provider, patient, and family across the illness and care trajectory; condition-specific education and support for family wellbeing; consideration of the family context, including strengths and cultural values; and the need for dedicated policies and procedures to support implementation of family-centred service models. Evidently there are many components underpinning family-centred service, and it is essential to consider how these components can be best implemented in the context of pABI rehabilitation.

#### Clinical implementation of family-centred service in paediatric ABI rehabilitation

Family-centred service has predominantly been investigated in the context of paediatric disability; however, most of this literature has focused on implementation in relation to congenital or developmental disabilities which are present “from the beginning” of a child's life (54). Although the key premises and elements of family-centred service are applicable and important in the care of children with pABI, rehabilitation focuses on children's recovery, whereas the “habilitation” of children with congenital or acquired disabilities focuses on optimising developmental gains (148). pABI also presents unique challenges to family adaptation due to its sudden and unforeseen nature, thus the implementation of family-centred service in this context must consider and attend to the unique needs of these families.

Limited literature has investigated the implementation of family-centred service in pABI rehabilitation. In a recent qualitative study, Botchway and colleagues (149) investigated rehabilitation models of care across a range of services, with a focus on family-centred service, psychosocial support, and transition periods. They described variability in approaches to delivering family-centred service, such as services hosting regular family meetings, providing tailored care to families, supporting families in care coordination, and engaging families in collaborative decision making. In keeping with previous literature, they also reported several barriers to family-centred service implementation, including insufficient funding and difficulty managing family dynamics, preferences, and expectations (7, 150). While family-centred service was often valued and included in services' models of care, the extent and success with which this approach was applied was often not evaluated. This suggests that services may have a limited understanding of the effectiveness of their current approaches and associated outcomes, aligning with previous literature that highlighted the need for regular evaluation of family-centred service processes to identify what is effective, and what requires modification (149, 150).

### Clinical implications

There is growing evidence that involving and supporting families in pABI rehabilitation can lead to improved outcomes for children and families. This poses significant implications for clinicians working in this context (Table 1). Clinicians work together in collaboration *with* children and families as “advocates, knowledge brokers, collaborators, facilitators, educators and coaches” (105), “rather than just doing ‘*to*’ or ‘*for*’ them.” (95). This active role requires clinicians to be equipped with skills in two-way communication and active listening, co-constructed engagement, assessing family needs, addressing psychological challenges, negotiating child and family voices and priorities, and tailoring rehabilitation to families' unique circumstances, strengths, priorities, cultures, and values across the entire care trajectory (7, 35, 85, 90, 102, 106, 142, 149, 151–154). It is essential that clinicians are provided with adequate opportunities to develop these skills and competencies, both during their training and through ongoing professional development. Increased competence in these skills can enable clinicians to better meet families' needs and support their participation, engagement, and attendance in rehabilitation, which may in turn improve children's and families’ outcomes following pABI.

**Table 1:** Key recommendations and actions for clinicians.

Recommendation	Relevant literature	Action
Develop a holistic understanding of the child and family and what is important to them, and tailor rehabilitation accordingly	D’Cruz et al. (2017)	• Use the Children and Youth Version of the ICF • Use the “F-words” • Use a narrative storytelling approach to support children and families to share individual and family stories, e.g. through spoken storytelling, story writing, drawing, collage, photography, song writing • Show interest in the child's interests, e.g., favourite sport team, hobbies • Tailor rehabilitation exercises and activities to child's interests, e.g., incorporate their favourite sport into physiotherapy sessions • If the child is unable to express their interests, ask their family and tailor rehabilitation accordingly • Make information and activities meaningful and relevant to children's and families’ unique lives
	Doering et al. (2011)	
	Jenkin et al. (2022)	
	Rosenbaum & Gorter (2012)	
	World Health Organization (2007)	
Develop an understanding of family needs	Gan & Wright (2019)	• Use formal measures to assess family needs, e.g., Family Assessment Device, Family Needs Questionnaire – Paediatric Version • Embed opportunities for sharing family needs into rehabilitation, e.g., during initial assessments, goal setting, discharge planning • Encourage involvement of all family members, including fathers and siblings, through developing a family-friendly environment (e.g., by displaying brochures depicting images of family members) • Understand the ways that families can be impacted by pABI and share examples to normalise families’ experiences • Discuss family needs and check in with families over time, especially at key transition points (e.g., school transitions) • Recognise “where the family is at” and ensure a balance between involving and supporting the family • Adopt a trauma-informed approach by providing early proactive and ongoing support • Link families with relevant supports, either within the rehabilitation service or externally (e.g., refer to community providers)
	Jenkin et al. (2022)	
	Kazak et al. (2006)	
	Miller et al. (1985)	
	Tully et al. (2021)	
Engage families in open communication and sharing	Bright et al. (2015)	• Demonstrate active listening, e.g., through paraphrasing and summarising, eye contact, body language • Demonstrate empathy and trust • Validate and normalise families’ experiences and emotions • Provide sufficient time for individuals to respond to questions, both within and between rehabilitation sessions • Create a safe and comfortable space that encourages the child and family to communicate and share with the rehabilitation team
	Clark-Wilson & Holloway (2020)	
	Jenkin et al. (2020)	
	Jenkin et al. (2022)	
	Kuo et al. (2012)	
	Phoenix et al. (2019)	
	Pritchard-Wiart et al. (2022)	
Support children's input and involvement	Collins et al. (2021)	• Acknowledge children's voice and insight and strive to engage with them before deferring to a parent/caregiver • Provide sufficient time for the child to respond to questions • Use simplified language, e.g., yes/no questions, ask child to choose an option from a selection of possible responses • Ask questions directly to the child, e.g., by using their name and making eye contact • Use alternate forms of communication, e.g., adaptive communication devices, visual stimuli (images, photographs, drawings), and gesture (thumbs up/down, pointing) • Clarify whether information shared by family members aligns with child's experience • Use children's own language, e.g., goals defined in their own words • Create a safe space for the child to share their voice • Engage with families to “boost” engagement of child
	Jenkin et al. (2020)	
	Jenkin et al. (2022)	
	Pritchard et al. (2020)	
	Pritchard-Wiart et al. (2022)	
Adopt a strengths-based approach and foster “positive feelings” in families, including pride, relief, excitement, and hope	Gan et al. (2012)	• Acknowledge parents’/caregivers’ and siblings’ efforts • Discuss the child's progress with family members and link to rehabilitation goals, including “bigger picture” goals • Use a strength-based approach to emphasise the child's and family's resilience, strengths, and the skills and knowledge that families bring to rehabilitation
	Gan & Ballantyne (2016)	
	Gauvin-Lepage et al. (2016)	
	Jenkin et al. (2020)	
	Phoenix et al. (2019)	
	Spina et al. (2005)	
Empower families to decide their level of involvement, and balance perspectives of children and family members	Collins et al. (2021)	• Provide families with education about pABI, rehabilitation processes (e.g., goal setting, roles of disciplines, planning for transition home or to the community) • Check in with families over time to determine whether they would like to change their level of involvement • Discuss child's and families’ preferences regarding family involvement, particularly during adolescence • Acknowledge the need to balance voices of the child and family members and how this changes over time, particularly during adolescence • Collaborate with the child and family in negotiating their input and opinions • Validate input of both the child and their family members
	Jenkin et al. (2020)	
	Jenkin et al. (2022)	

True partnership is central to family-centred service, and it is the responsibility of clinicians to put this in place and “create space for the voice of the family to be properly heard” (35, 155). Although family-centred service represents a shift, rather than a major departure, from previous healthcare, the change in power relations that is characteristic of family-centred service may lead to clinicians feeling threatened, devalued, and unskilled (7, 152). Clinicians may feel that they lack competence and confidence to practice in a family-centred way (7, 88, 152), and may instead feel more comfortable adhering to a more traditional, biomedical model of service delivery (7). These concerns may be alleviated by increasing the focus of clinicians' training and professional development on family-centred service, and emphasising the role of clinicians in skilfully involving families in rehabilitation (152).

The implementation of family-centred service requires “innovation at multiple levels” (151), including at individual, service, and policy levels. In addition to supporting implementation through clinician training and education, it is essential that family-centred service is implemented at a service level. Professionals within organisations that emphasise family-centred values are more willing to embrace a family-centred approach, and clinicians are more willing to accept new practice approaches when guided by managers and leaders (88, 156). Importantly, rehabilitation services should strive to better articulate what they mean by “family-centred service” within their models of care, particularly given the documented variability in definitions and service approaches (9, 149, 157). This may be facilitated through the development of evidence-based best practice guidelines and targeted interventions at individual, service, and policy levels.

### Future research directions

Family-centred service literature stresses that families are different and unique and that understanding family contexts is central to family-centred service delivery; however, the best approach for gaining and applying this understanding in pABI rehabilitation remains unclear. The Children and Youth Version of the ICF (158) and “F-words” (85) provide models that can assist clinicians to develop a holistic understanding of the child and their family, but have not yet been investigated in the context of pABI. Optimally clinicians should develop an understanding of, and address, family needs across the care trajectory following pABI. Although tools have been developed to assess family functioning and needs [e.g., Family Needs Questionnaire – paediatric version (159), Family Assessment Device (160)], their usefulness in pABI rehabilitation remains unclear. In developing an understanding of family needs following pABI, it is important to consider how to address families' changing needs across the care trajectory. For example, trauma-informed approaches to care may be particularly important during early, sub-acute rehabilitation to support family adaptation and coping. In contrast, later rehabilitation may focus on developing an understanding of families' unique lives and contexts to maximise children's participation in school and the community. It is therefore essential that service delivery is tailored to families' changing needs across the care trajectory and the child's developmental stages, highlighting the complexity of delivering family-centred service within the context of pABI rehabilitation.

In addition to considering family needs during rehabilitation, it would be beneficial to develop resources to support clinicians' understanding of family interests and values. There are no existing measures that assess child and family values within healthcare settings, thus this represents an area for future research innovation. Given that time is a key barrier to implementing family-centred service within rehabilitation, it is important that key stakeholders, including clinicians, are actively involved in such research efforts to ensure that such resources and tools can be feasibly implemented in practice. The use of such resources and tools would enable clinicians to adapt service delivery to families' unique needs, preferences, and values, thus enhancing family-centred service delivery.

Family participation, attendance, and engagement in children's healthcare represent under-investigated elements of family-centred service. While a recent qualitative study outlined the ways that families participate in pABI rehabilitation (107), further investigation is required to determine how clinicians and services can better support family participation and engagement in rehabilitation. For example, collaborative goal setting represents a key opportunity for families to have input into their children's healthcare. It is therefore essential that clinicians working in pABI rehabilitation develop and enhance their skills in collaborative goal setting, for example, through training packages. Providing psychoeducation and evidence-informed resources to families to support their understanding of, and engagement in, collaborative goal setting, is also key to empowering them to be actively involved in this process. Greater consideration must also be given to the participation, attendance, and engagement of family members in rehabilitation. Literature investigating family needs following pABI typically focuses on mothers' perspectives, and fathers are often under-represented in parent-focused interventions for child mental health (161–165), with common barriers including work commitments and a lack of time (166). Flexible service delivery (e.g., face-to-face and online options) is therefore essential to support fathers' engagement in children's healthcare (167). While literature focusing on family participation in children's healthcare typically focuses on parents and caregivers only, future research should also consider siblings' needs and participation.

Family-centred service is considered best practice in pABI rehabilitation, yet there remains no evidence-based clinical practice guidelines to support implementation. Given that rehabilitation tailored to children's and families' dynamic needs and preferences is essential, future research in this context must involve early, ongoing, and meaningful engagement of children with pABI and their families in study design, implementation, and evaluation to optimise feasibility and implementation of findings into clinical practice. Partnership with families, including in future research efforts, is an important step towards further improving aspects of family-centred service in pABI rehabilitation, and requires skills in family engagement across all stages of the research process. Using co-design methods to develop and evaluate training packages that support clinicians and researchers in enhancing their skills in family-centred service will also be important. Given that there is currently limited research that has investigated the effectiveness of aspects of family-centred service post-pABI, future research should aim to better understand the application of components of family-centred service in paediatric ABI rehabilitation to ensure that the unique needs of children with ABI and their families are considered.

## Conclusions

Working with children with pABI and their families in rehabilitation is complex, and there is growing evidence for a family-centred approach addressing child and family needs. Despite this, there remains a lack of clarity and evidence-based guidance about how family-centred service should be delivered in pABI rehabilitation. Current literature across pABI rehabilitation and family-centred service demonstrates the active role of clinicians in implementing family-centred service and underscores the importance of family-centred approaches being adopted at the level of services' models of care. This review highlights the need for ongoing, collaborative research efforts to inform development of evidence-based guidelines for the implementation of family-centred service in pABI rehabilitation.
